# Dynamic Mathematical Modelling of the Removal of Hydrophilic VOCs by Biotrickling Filters

**DOI:** 10.3390/ijerph120100746

**Published:** 2015-01-14

**Authors:** Pau San-Valero, Josep M. Penya-roja, F. Javier Álvarez-Hornos, Paula Marzal, Carmen Gabaldón

**Affiliations:** Research Group GI^2^AM, Department of Chemical Engineering, University of Valencia, Avda. Universitat s/n, 46100 Burjassot, Spain; E-Mails: pau.valero@uv.es (P.S.); josep.penarrocha@uv.es (J.M.P.); Francisco.j.alvarez@uv.es (F.J.A.-H.); paula.marzal@uv.es (P.M.)

**Keywords:** biotrickling filtration, air pollution control, volatile organic compounds, mathematical modelling

## Abstract

A mathematical model for the simulation of the removal of hydrophilic compounds using biotrickling filtration was developed. The model takes into account that biotrickling filters operate by using an intermittent spraying pattern. During spraying periods, a mobile liquid phase was considered, while during non-spraying periods, a stagnant liquid phase was considered. The model was calibrated and validated with data from laboratory- and industrial-scale biotrickling filters. The laboratory experiments exhibited peaks of pollutants in the outlet of the biotrickling filter during spraying periods, while during non-spraying periods, near complete removal of the pollutant was achieved. The gaseous outlet emissions in the industrial biotrickling filter showed a buffered pattern; no peaks associated with spraying or with instantaneous variations of the flow rate or inlet emissions were observed. The model, which includes the prediction of the dissolved carbon in the water tank, has been proven as a very useful tool in identifying the governing processes of biotrickling filtration.

## 1. Introduction

Control of volatile organic compound (VOC) emissions from industry is nowadays a priority in air quality regulation. In the European Union (EU), Directive 1999/13/EC, recently modified by Directive 2010/75/EU, pursues the reduction of VOC emissions. According to data from the European Environment Agency, the sector of “solvents and product use” contributes 44% of the emissions of non-methane VOC in the EU; a reduction of 42% from 1990 to 2012 has been reported with a release of 2951 Gg in 2012 [1]. The industrial use of solvents typically releases waste gas streams where the flow is high (>1000 m^3^·h^−1^) and the VOC concentration is low (<5 g·m^−3^). Bioprocesses are best suited to the control of these emissions due to the low concentration of pollutants [2]. Among bioprocesses, biotrickling filtration is recommended for compounds with Henry’s law constants below 0.1 [3], such as ethanol, n-propanol or isopropanol, the main pollutants of the waste gas streams emitted from flexible food packaging industries (flexographic sector). A biotrickling filter (BTF) consists of a column filled with an inert packing material, where the biomass attaches to the media and develops a biofilm. In this configuration, the gas and liquid phases circulate through the column in co- or counter-current mode. Depending on the operation mode, the BTF process involves a continuous or intermittent trickling of water. 

Despite the fact that BTFs have been successfully applied for the treatment of air pollutants, the use of BTFs depends on the increase of operational knowledge to allow the robustness of the performance. As has been recognized, the performance of BTF is markedly dependent on the operational conditions. Parameters, such as liquid velocity, gas velocity and empty bed residence time (EBRT) and inlet concentrations, may hinder the performance of field-scale BTFs [4,5,6]. Thus, further research for a better understanding of the role of those parameters would be desirable [7]. Several efforts have been made to adapt the operational conditions of laboratory experiments to emulate the operational conditions usually experienced in industrial applications [6]. One of the most common practices in industrial BTFs in comparison with laboratory studies is the use of intermittent trickling of water. San Valero *et al.* [8] observed that a spraying regime of 15 min every 1.5 h resulted in peaks of concentration coinciding with the irrigation of the bed when treating isopropanol. We concluded that the frequency of irrigation is a crucial parameter in terms of the achievement of low emissions under intermittent loading of highly-soluble compounds.

Biotrickling filtration involves a complex combination of several physical, chemical and biological processes; further investigation, in order to integrate the phenomena occurring during treatment, is required. Mathematical modelling is a fundamental tool in the development of an understanding of the process. Additionally, as pointed out by Lu *et al.* [9], effective modelling can lead to the development of a trustworthy performance equation that decreases the time and cost of experimentation on the pilot scale. Thus, phenomenological models based on the main mechanisms on biofiltration seem to be useful in improving the understanding of BTFs and in identifying the governing processes involved in their operation. The phenomenological model most commonly used during the last few decades was developed by Ottengraf and Van Den Oever [10] for biofilters operating under steady-state conditions. Following this study, many models for biofilters have been reported in the literature, adding new phenomena, such as adsorption in the packing material and the inhibition kinetics of microbial growth, among others [11]. As an example, Shareefdeen *et al.* [12] included both oxygen and substrate inhibition effects; this model was improved by assuming the partial coverage of the support particles by biofilm and by modelling the adsorption on the uncovered particles by using the Freundlich isotherm [13]. 

Most mathematical models of BTFs and trickle bed biofilters are based on mechanisms that have been used to describe biofilter behavior. Usually, a three-phase model (gas-liquid-biofilm) is used to describe these configurations. The main difference with biofilter models is related to the presence of a liquid phase, often simulated as an intermediate step between the gas phase and the biofilm. In steady state conditions, Mpanias and Baltzis [14] were the first to develop a model that takes into account the kinetic limitations arising from the availability of VOCs and oxygen in BTF. This model was extended to VOC mixtures [15]. Concerning non-steady state conditions, Okkerse *et al.* [16] presented a dynamic BTF model for the degradation of volatile acidifying pollutants, as well as mass accumulation and mass distribution along the column. Zhu *et al.* [5] proposed a dynamic three-phase system with a non-uniform bacterial population to show the effect of mass transfer limitations due to the water phase in the reactor for the removal of diethyl ether. Kim and Deshusses [17] developed a dynamic BTF model for the removal of H_2_S with gas and liquid phase flowing counter-current, where the biofilm on the packing material was not completely wetted. In this model, a fraction of the pollutant was transferred directly from the gas phase to the biofilm, while another fraction was transferred through the liquid phase to the biofilm. Recently, Almenglo *et al.* [18] modified the Kim and Deshusses model by assuming a stagnant liquid fraction distributed homogeneously along the bed; mass balances in the biofilm were divided into “flowing” biofilm (which is in contact with flowing liquid) and “stagnant” biofilm (which is in contact with stagnant liquid). Lee and Heber [19] proposed a model modified from that of Alonso *et al.* [20] in order to develop a genetic algorithm to estimate the unknown parameters in ethylene removal. Iliuta *et al.* [21] developed a predictive dynamic model in a trickle-bed bioreactor based on the macroscopic volume-averaged mass and momentum balance equations coupled with classical diffusion and bioreaction equations to illustrate the influence of biomass accumulation on a bioreactor for toluene removal. These authors showed that shifting from a biofilter to a trickle-bed bioreactor reduces the removal efficiency, due to an extra liquid-film mass transfer resistance step. However, despite the fact that these models provide valuable information on the understanding of the behavior of bioreactors, there is still a lack of information with respect to models adapted to industrial emissions characterized by an intermittent spraying pattern, variable gas flow rate and variable inlet concentration. In this regard, more effort is required to obtain more realistic simulations and to identify the main differences in the observed behavior between the laboratory scale and the industrial scale. As was pointed out by Devinny and Ramesh [22], no single model has become generally accepted. 

The aim of the present research is to go deeper into the intricacies of the treatment of hydrophilic compounds using biotrickling filtration technology. For this purpose, a mathematical model was developed to simulate the performance of BTFs, taking into account the main operational conditions found in the industry. The model presented herein was prepared for simulating systems with complex inlet concentration patterns, gas flow rates and cyclic conditions of spraying. An intermittent spraying regime implies that the liquid phase varies during the filter operation, making it necessary to distinguish two different situations, corresponding to spraying and non-spraying periods. In addition, the operation of BTF usually requires more than one spraying pattern during the same day related to periods with different feeding conditions or clogging control, among others. In this regard, the model is able to combine these two different patterns.

The following objectives have been achieved: (1) developing a dynamic mathematical model based on different behaviors during spraying and non-spraying periods combined with variable inlet concentration and variable gas flow rate; (2) calibrating and validating the model with data from BTF at the laboratory scale using isopropanol as the target pollutant; and (3) validating the model with data from a BTF located in an industrial facility from the flexographic sector. 

## 2. Experimental Section 

### 2.1. Experimental Set-Up and BTF Operational Conditions for the Experiments at Laboratory Scale 

Two sets of independent data were used to test the mathematical model. The first set corresponds to data from laboratory experiments using isopropanol as the target pollutant. The experiments were performed using a laboratory-scale BTF composed of three cylindrical methacrylate modules in series, with a total bed length of 100 cm and an internal diameter of 14.4 cm. The BTF was filled with a random packing material of 25 mm in diameter (Flexiring: superficial area (*a*) = 207 m^2^·m^−3^; porosity of the packing material (*θ_PM_*) = 0.92), using a volume of the bed of 16.32 L. The BTF had 20 cm of free spaces at the top and bottom of the column and was equipped with 3 equidistant sampling ports. The stream was contaminated with isopropanol, which was introduced through the bottom of the column. A recirculation solution was fed into the bioreactor in counter-current mode with respect to the air flow using a centrifugal pump at 2.5 L·min^−1^ from a tank with 3.5 L of solution. An air stream polluted with VOCs was supplied to the BTF for 16 h per day (starting at 8:00 am), while the rest of the time (8 h), the BTF was supplied with clean air, maintaining a constant air flow rate. A BTF inoculated with activated sludge ran for more than 3 months with an inlet load (IL) of 30 g–C·m^−3^·h^−1^, EBRT of 60 s and intermittent spraying. With this IL, two consecutive intermittent spraying patterns (Run 1 and Run 2) were applied during a minimum of a 2-week period. Table 1 summarizes the performance of the system during the last 5 days (from Monday to Friday) of each of the runs (1 and 2). Then, the inlet load was increased to 60 g–C·m^−3^·h^−1^ for 2 weeks. Run 3 (Table 1) summarizes the performance of the system during the final week (from Monday to Wednesday). Each run started with clean water in the recirculation tank. No purges were undertaken until the end of each run. The pressure drop was negligible (<1 Pa·m^−1^). The liquid hold-up (*θ_L_*) had an average value of 0.093 ± 0.003. The biofilm content was gravimetrically determined (mass contained in two rings extracted from each of the three sampling ports). The volume fraction of the biofilm (*θ_B_*) calculated from the biofilm content of the bioreactor (specific gravity of 1) resulted in a value of 0.18 ± 0.04. The water content of the biofilm was measured as 95 ± 3%; 50 kg·m^−3^ of biomass concentration (*X_V_*), which was selected as the average value for modelling purposes.

**Table 1:** Overall performance of the laboratory-scale experiments. EBRT, empty bed residence time.

	Inlet load ^a^ (g–C·m^−^^3^·h^−^^1^)	EBRT (s)	Spraying Pattern	Elimination Capacity ^a^ (g–C·m^−^^3^·h^−^^1^)	Removal Efficiency ^a^ (%)
Run 1	32.6	60	1 h every 4 h	29.8	91
Run 2	32.0	60	30 min every 4 h	29.7	93
Run 3	59.6	60	1 h every 4 h	53.6	90

^a^ Average value from periods during pollutant feeding (16 h·d^−1^).

### 2.2. Experimental Set-Up and BTF Operational Conditions for the Field Scale 

Data from an industrial BTF (VOCUS^TM^, PAS Solutions BV, the Netherlands) located at a flexographic industry site were used. The waste gas stream was composed of a mixture of VOCs (63% ethanol, 22% ethyl acetate, 13% 1-ethoxy-2-propanol). The manufacturing shift of this industrial site was 18 h of emissions per day (from 6 to 24 h) during 5 days a week (working days), with several emission sources. The BTF system consisted of a packed reactor with a volume of 49 m^3^ filled with polypropylene rings (Flexiring 50 mm: *a* = 102 m^2^·m^−3^; *θ_PM_* = 0.93), plus a recirculation tank with a maximum water capacity of 15 m^3^. The bioreactor was operated in counter-current mode; air from the factory was blown into the bottom of the column continuously (polluted air for 18 h per day and clean air for 6 h per day). The BTF began operating in June 2009. Two sets of data (from Monday to Thursday) with different spraying frequency (October 2009, and January 2011) were selected (Table 2). During weekends, clean air was blown on the BTF, and the spraying frequency was kept the same as those applied during non-VOC feeding periods in working days in order to promote the removal of the accumulated VOCs in the water tank. Volumes of water in the recirculation tank were 6.5 and 12.8 m^3^ (25% of the volume renewed with fresh water on Sundays), and the liquid flow rates were 30 and 32 m^3^·h^−1^ for Run 4 and Run 5, respectively. The pressure drop was lower than 15 Pa·m^−1^. The system worked for more than 3 months under each of the selected conditions. Table 2 summarizes the performance of the system during the 4 days of each run. The volume fraction of the mobile liquid phase in the bioreactor was approached by measuring the quantity of accumulated water in the bioreactor during spraying; a value of 0.05 was obtained (*θ_L_*).

**Table 2:** Overall performance of the industrial biotrickling filter (BTF).

	Date	Inlet Load ^a^ (g–C·m^−3^·h^−1^)	Gas Flow Rate ^a ^(m^−3^·h^−1^)	Spraying Pattern		Elimination Capacity ^a^ (g–C·m^−3^·h^−1^)	Removal Efficiency ^a^ (%)
				0–6 am	6 am–12 pm		
Run 4	October 2009	27.5	1675	6 min every 21 min	6 min every 21 min	17.9	65
Run 5	January 2011	46.5	2717	6 min every 1 h	6 min every 3 h	29.1	63

^a ^Average value from periods during pollutant feeding (18 h·d^−1^).

### 2.3. Analytical Methods 

In the laboratory experiments, the gas concentration of isopropanol was measured using a total hydrocarbon analyzer (Nira Mercury 901, Spirax Sarco, Spain). The response factor of the total hydrocarbon analyzer was determined by gas chromatography (model 7890, Agilent Technologies, USA). The determination of the total organic carbon (TOC) in water was measured using a total organic carbon analyzer (TOC-VCHS, Shimadzu Corporation, Japan). At the industrial scale, the inlet and outlet gas VOC concentrations were continuously monitored using a total hydrocarbon analyzer (RS 53-T, Ratfisch Analysensysteme GmbH, Germany). The air flowed during the whole day, and the air flow rate was monitored continuously by using a pitot tube (19″ pitot tube, Testo AG, Germany).

### 2.4. Model Development 

**Figure 1:**
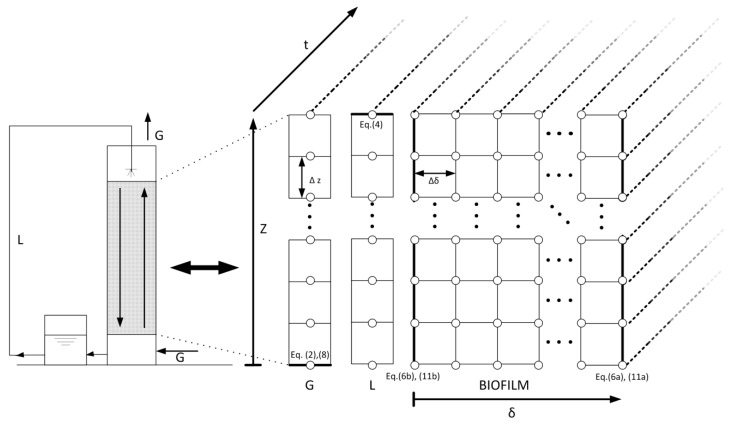
Conceptual scheme of the BTF and model derivation. Bold lines indicate the boundary conditions. Eq. means equation.

The model was based on the fact that BTFs are usually operated in intermittent spraying mode. The intermittent spraying regime implies two different behaviors of the system. Thus, a mobile liquid phase and a stagnant liquid phase were considered during the spraying period and non-spraying period, respectively. Thus, a three-phase model (gas-liquid-biofilm) is proposed here based on the mass balances of the gas and liquid phases and the biofilm. Two different systems of partial differential equations were used, depending on whether the spraying or the non-spraying period was simulated. The conceptual scheme of the BTF and the model derivation are shown in Figure 1. For model development, the following assumptions were made:

(1) The gas phase flows in a plug flow regime along the filter bed.
(2) Axial dispersion is neglected.
(3) The adsorption of pollutant in the packing material is negligible.
(4) The active biofilm is formed on the external surface of the packing material, and no reaction occurs in the pores. The biofilm covers the surface of the packing material, and its thickness (δ) is much smaller than the size of the solid particles, so a planar geometry is assumed.
(5) The packing material is completely covered by the biofilm.
(6) The biodegradation kinetics are described by a Monod expression, indicating the oxygen limitation.
(7) The diffusion inside of the biofilm is described by Fick’s second law.
(8) A mobile liquid phase is assumed during the spraying period, and a stagnant liquid phase is considered during the non-spraying period.
(9) The gas-liquid interface is in equilibrium according to Henry’s law.
(10) The mass flux at the gas-liquid and the liquid-biofilm interfaces can be expressed by mass transfer coefficients.
(11) The presence of biomass in the bioreactor increases resistance to the mass transfer between the gas and the liquid phase. Thus, the overall mass transfer coefficients experimentally determined in abiotic conditions are corrected by a factor (*α_1_*) varying between 0 and 1.
(12) There is no reaction in the liquid phase.

According to the assumptions made above, the mass balances during both periods are described in the next subsection.

#### 2.4.1. Mass Balances during the Spraying Period

During the spraying period, the pollutant and the oxygen are transferred from the gas phase to the mobile liquid phase and then to the biofilm, where the biodegradation takes place. The mass balance of the gas phase is described according to Equation (1) for a system in counter-current operation:

(1) $$\theta_{G}\frac{\partial C_{G_{i}}}{\partial t}=-v_{G}\frac{\partial C_{G_{i}}}{\partial z}-\alpha_{1}K_{L}a_{i}\left(\frac{C_{G_{i}}}{H_{i}}-C_{L_{i}}\right)$$

where *i* denotes the pollutant (*P*) or the oxygen (*O*). Thus, for component *i*, *C_G__i_* and *C_Li_* are the concentrations of gas and liquid phases, respectively; *K_L_a_i_* is the overall mass transfer coefficient; *H_i_* represents the dimensionless Henry’s law constants expressed as the concentration of the gas phase/concentration of the liquid phase; *t* denotes time; *z* is the distance from the bottom of the column; *v_G_* is the superficial air velocity; and *θ_G_* is the porosity of the bed calculated as *θ_G_* = *θ_PM_* – *θ_L_* – *θ_B_*.

The boundary condition of Equation (1) is given by:

(2) $$z=0\;C_{G}=C_{G_{i}}^{in}$$

where $C_{G_{i}}^{in}$ is the inlet concentration of the component *i*.

The mass balance of the mobile liquid phase is given by:

(3) $$\theta_{L}\frac{\partial C_{L_{i}}}{\partial t}=v_{L}\frac{\partial C_{L_{i}}}{\partial z}+\alpha_{1}K_{L}a_{i}\left(\frac{C_{G_{i}}}{H_{i}}-C_{L_{i}}\right)-\frac{D_{i}a}{\beta }(C_{L_{i}}-S_{i,1})\;$$

where for component *i*, *D_i_* is the diffusion coefficient in water; *S_i,_*_1 _is the concentration in the biofilm interface; *v_L_* is the superficial liquid velocity; and *β* is the thickness of the liquid-biofilm interface.

The boundary condition of Equation (3) is given by the mass balance in the recirculation tank:

(4) $$z=Z\;\frac{\partial C_{L_{i}}}{\partial t}=\frac{Q_{L}}{V_{T}}(C_{L_{i_{z=0}}}-C_{L_{i_{z=Z}}})$$

where *Z* is the height of the column; *Q_L_* is the liquid flow rate; and *V_T_* is the volume of the recirculation tank. *C_Li_* *_z=0_* and *C_Li_* *_z=Z_* are the concentrations of the component *i* in the liquid phase at the bottom and top of the column, respectively.

The mass balance of the biofilm is given by:

(5) $$\frac{\partial S_{i}}{\partial t}=f(X_{v})D_{i}\frac{\partial^{2}S_{i}}{\partial x^{2}}-\frac{\mu_{\max}X_{v}}{Y_{i}}\frac{S_{P}}{S_{P}+K_{P}}\frac{S_{O}}{S_{O}+K_{O}}$$

where $S_{i}$ is the concentration inside the biofilm of component *i*; $f(X_{v})$ is the correction factor of the diffusivities in water due to the biomass calculated by Fan’s equation [23]; $X_{v}$ is the concentration of the biomass; $\mu_{\max}$ is the specific growth rate of the biomass; $K_{P}$ and $K_{o}$ are the half-saturation constants of the pollutant and oxygen, respectively; and, for component *i*, $Y_{i}$ is the yield coefficient. 

The boundary conditions for the mass balance of the biofilm are given by:

(6a) $$x=\delta \;\frac{\partial S_{i}}{\partial x}=0$$

(6b) $$x=0\;S_{i}=C_{L_{i}}$$

where *δ* is the biofilm thickness.

#### 2.4.2. Mass Balances during the Non-Spraying Periods

During the period without spraying, the pollutant and the oxygen are transferred from the gas phase to the stagnant liquid phase and then to the biofilm, where biodegradation takes place. The according mass balances are presented below.

The mass balance of the gas phase is given by Equation (7):

(7) $$\theta_{G}\frac{\partial C_{G_{i}}}{\partial t}=-v_{G}\frac{\partial C_{G_{i}}}{\partial z}-\alpha_{2}\alpha_{1}K_{L}a_{i}\left(\frac{C_{G_{i}}}{H_{i}}-C_{L_{i}}\right)$$

where *α_2_* is a switch parameter that weights the gas-liquid mass transfer resistance. A value of 100 is assumed to indicate that the gas-liquid mass transfer resistance is negligible. A value of 1 indicates that a resistance to the gas-liquid mass transfer contributes to reducing the pollutant max flux diffusing to/from the biofilm. 

The boundary condition of Equation (7) is given by:

(8) $$z=0\;C_{G}=C_{G}^{in}$$

The mass balance of the stagnant liquid phase is given by:

(9) $$\theta_{L}\frac{\partial C_{L_{i}}}{\partial t}=\alpha_{2}\alpha_{1}K_{L}a_{i}\left(\frac{C_{G_{i}}}{H_{i}}-C_{L_{i}}\right)-\frac{D_{i}a}{\beta }(C_{L_{i}}-S_{i,1})\;$$

The mass balance in the biofilm is given by:

(10) $$\frac{\partial S_{i}}{\partial t}=f(X_{v})D_{i}\frac{\partial^{2}S_{i}}{\partial x^{2}}-\frac{\mu_{\max}X_{v}}{Y_{i}}\frac{S_{P}}{S_{P}+K_{P}}\frac{S_{O}}{S_{O}+K_{O}}$$

with the boundary conditions:

(11a) $$x=\delta \;\frac{\partial S_{i}}{\partial x}=0$$

(11b) $$x=0\;S_{i}=C_{L_{i}}$$

#### 2.4.3. Numerical Solution

The partial differential equations given above constitute two second-order nonlinear distributed systems. In order to solve them, the method of lines (MOL) was chosen. For a numerical problem solution, *Z* is divided into *N* sections with *N* + 1 equi-spaced node points. Similarly, the biofilm thickness is divided into *M* sections with *M* + 1 points, resulting in two ordinary differential equation (ODE) systems of 2(*N* + 1)(*M* + 3) equations. The next step is to discretize the spatial variables. The parameters of *N* and *M* were optimized as 20 and 40, respectively. The resulting ODE systems were found to be stiff. Therefore, they were integrated using the ode23t function of Matlab.

## 3. Results and Discussion

### 3.1. Model Calibration 

**Figure 2:**
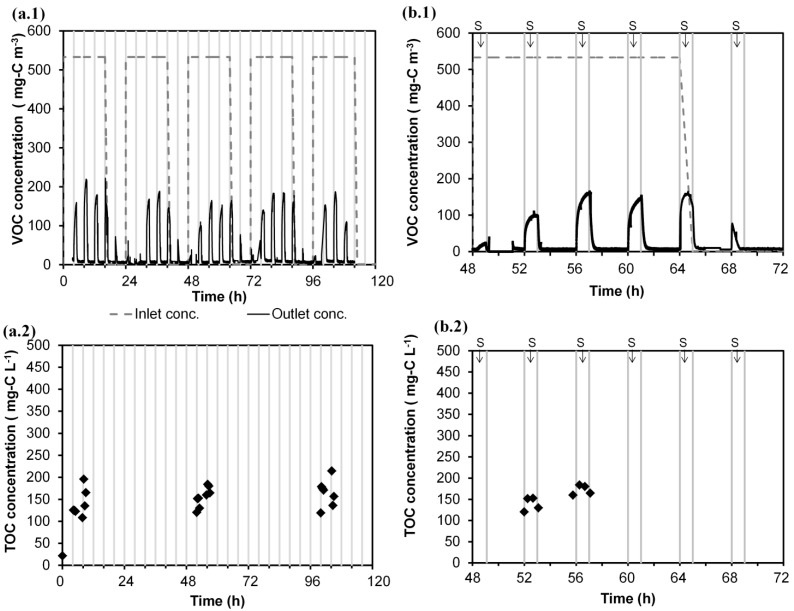
Experimental data of Run 1 (Monday to Friday), where each central line denotes a spraying: (**a.1**) concentration of VOC in the gas phase; (**a.2**) concentration of the dissolved carbon in the water tank. Wednesday results, where S denotes the spraying periods: (**b.1**) concentration of VOC in the gas phase; (**b.2**) concentration of the dissolved carbon in the water tank.

The calibration of the mathematical model presented herein was carried out with the experimental Run 1 (Table 1). Figure 2 shows the monitoring of the pollutant concentration in the gas phase (a.1) and the evolution of the dissolved carbon concentration in the water tank (a.2) from Monday to Friday (0 to 120 h). In Figure 2 (a.1 and a.2), time 0 h refers to Monday 8:00 am. As an example of the daily patterns, data for Wednesday (48 to 72 h) is shown in Figure 2 (b.1 and b.2). As is shown in Figure 2 (a.1 and b.1), the outlet gas stream exhibited peaks of pollutant emission coinciding with the spraying periods (six peaks per day). During the periods without spraying, nearly complete removal of the pollutant was obtained. For every day, a similar dynamic pattern was observed. During the feeding of air polluted with VOC (0–16 h), the pollutant leak associated with the first spraying of each day (0 h) was much lower than the rest of the peaks. The leak increased during the second spraying (4 h), reaching a quasi-stable maximum during the third and fourth spraying (8 h, 12 h). After cutting off the supply of VOC (at 16 h), the immediate peak (16 h) was similar to those obtained previously, but after some hours, the outlet gas emission during spraying was drastically reduced (20 h). The evolution of the peaks during the non-VOC feeding period indicated that the accumulated substrate in the system was consumed; the gas-phase leak at 20 h of each day was nearly in equilibrium with the organic liquid concentration in the tank. The dissolved carbon concentration in the water tank was monitored during spraying at working hours on alternate days (Figure 2 (a.2, b.2)). As can be seen, the daily concentration increased with each spraying, indicating that the pollutant is absorbed in water. The outlet gas emissions of the second to fifth daily peaks were more than three-times higher than the predicted equilibrium concentration with the dissolved carbon concentration in the water. The variations of the liquid concentration in the recirculation tank between days indicated that the accumulated pollutant in water would be degraded in the BTF during spraying in night periods (16–24 h). 

The parameters for the calibration of the model are summarized in Table 3. The values of physical constants, diffusivities in water and Henry’s law constants have been taken from the literature. The overall mass transfer coefficients of the abiotic system were estimated using the experimental correlations proposed in San-Valero *et al.* [24] for the operational conditions used in the present work. Biofilm thickness was assumed to be 60 μm. The yield coefficient of the isopropanol was taken from the literature, and the yield coefficient of the oxygen was calculated using the stoichiometric balance. 

**Table 3:** Parameters used in the modelling of BTF at the laboratory scale.

Parameters	Specific Value	Reference
Physical properties		
*D_P_ (m^2^*·*s*^−*1*^*)*	1.13 × 10^−9^	[25]
*D_O_ (m^2^*·*s*^−*1*^*)*	2.0 × 10^−9^	[26]
*H_P_*	2.8 × 10^−4^	[24]
*H_O_*	31.4	[27]
*K_L_a_P _(s* ^−*1*^ *)*	2.98 × 10^−5^	Using correlation in [24]
*K_L_a_O _(s* ^−*1*^ *)*	0.0126	Using correlation in [24]
Biofilm properties		
*δ (m)*	60 × 10^−6^	This work
*β (m)*	3.8 × 10^−6^	This work
Kinetic data		
*f(Xv)*	0.3495	[23]
*Ko*	0.26	[12]
*Y_P_*	0.48	[11]
*Yo*	0.14	Stoichiometric balance
*µ_max _(s* ^−*1*^ *)*	2 × 10^−5^	This work
*Ks_P_ (g–C*·*m*^−*3*^*)*	350	This work

**Figure 3:**
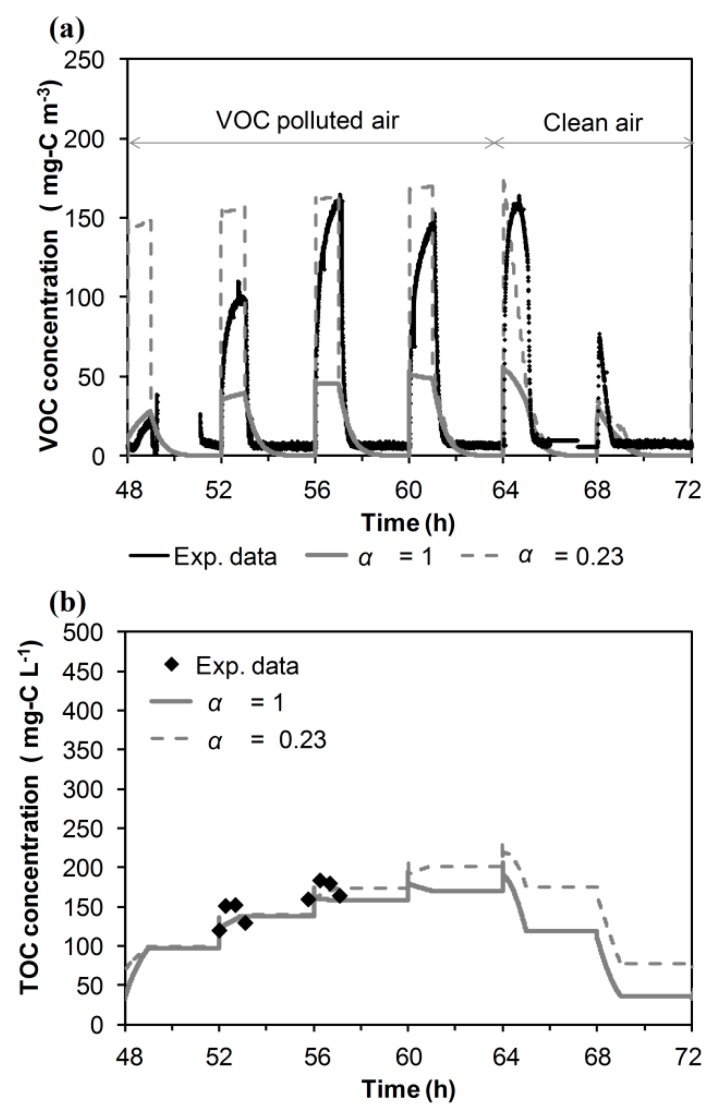
Influence of the parameter *α_1_* on the model predictions of Wednesday data (Run 1). (**a**) Outlet concentration of VOC in the gas phase. (**b**) Concentration of the dissolved carbon in the water tank.

With this set of parameters, the calibration process started by determining the values of the thickness of the liquid-biofilm interface (*β*) and the biokinetic parameters (*µ_max_*, *Ks_P_*) that predict the experimental evolution of the outlet concentration and the dissolved carbon in the water tank. During non-spraying, the mass transfer resistance between the gas and stagnant liquid was considered negligible (*α_2_* = 100), and during spraying, the gas-liquid mass transfer resistance was assumed to be equal to that determined under abiotic conditions (*α_1_* = 1). The value proposed for *µ_max_*, herein, 2 × 10^−5^ s^−1^, appears to be in agreement with the values obtained in the literature for the treatment of isopropanol. Bustard *et al.* [28] compiled data from different models proposed by different authors, with values ranging between 1.77 × 10^−5^ s^−1^ and 2.58 × 10^−5 ^s^−1^. The relative error deviation between experimental elimination capacity (EC) (29.8 g–C m^−3^ h^−1^) and simulated EC (31.8 g–C m^−3^ h^−1^) for Run 1 was 6.7%, indicating the feasibility of the model to reproduce the overall removal, although the dynamic pattern deviated from the experimental one. As an example, Figure 3 shows the results for Wednesday (48 to 72 h). The model predicts the existence of pollutant peaks associated with the mass transfer resistance of the gas-liquid film. The model predicts the sharp decrease of the gas-phase outlet concentration after spraying stops, corroborating that mass transfer resistance during non-spraying was negligible. The coupling of the spraying and non-spraying set of equations also predicts the periodical decrease of the dissolved carbon concentration in the water tank when the BTF works using clean air (from 64 to 72 h in Figure 3). This behavior is associated with the VOC desorption from the liquid to the gas phase and its biodegradation in the biofilm. However, the model underestimates the concentration of the outlet emissions in the case of the second to fifth peaks. In a second stage, the gas-liquid mass transfer flux was reduced by applying a correction factor in order to predict the maximum peak (*α_1_* = 0.23). As can be seen in Figure 3, this increase in the resistance to mass transfer overestimates the first two peaks after VOC feeding resumption after 8 h running with clean air. Experimental data indicated that the supply of VOCs, followed by long non-supply VOC periods (8 h per day), caused a cyclical variation in the resistance to mass transfer between the gas phase and liquid phase over time. This variation could be associated with a transient evolution in the physical properties due to biological reactions. For example, the accumulation of water and extracellular polymeric substances (EPS) could act as a periodical transfer barrier, deteriorating the removal efficiency of the system. When clean air was supplied, the accumulated VOC in the system could be biodegraded, and part of the formed EPS could disappear from the system, linked to the consumption by the microorganisms. In this regard, Zhang and Bishop [29] suggested that EPS could be used as a substrate and concluded that EPS was biodegradable by its own producers, as well as by other microorganisms, during periods without feeding of VOC. 

**Figure 4:**
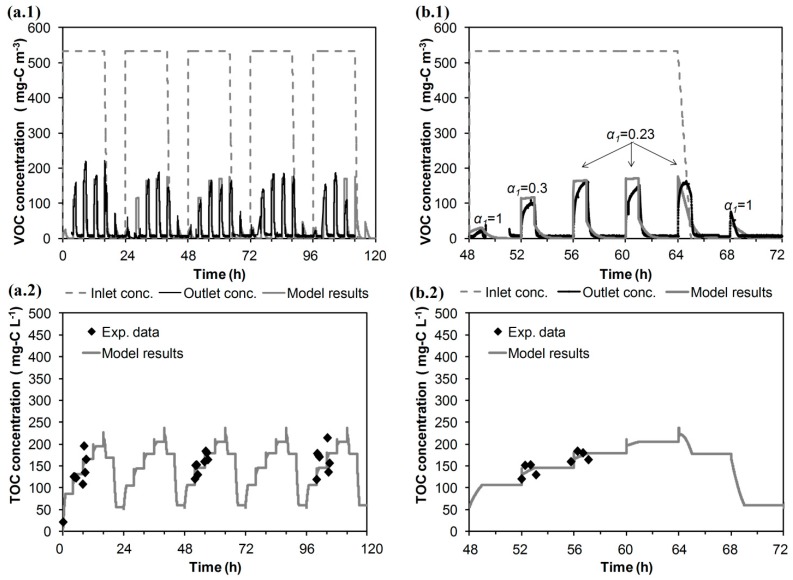
Weekly evolution of experimental data (Run 1) and model predictions for the treatment of isopropanol by a BTF. (**a.1**) concentration of VOC in the gas phase; (**a.2**) concentration of the dissolved carbon in the water tank. Wednesday results: (**b.1**) concentration of VOC in the gas phase; (**b.2**) concentration of the dissolved carbon in the water tank. Conc. means concentration and Exp. means experimental.

The calibration of the model ends by fitting the daily variation of the correction factor of gas-liquid mass transfer resistance over time. The same daily variation was assumed for the five days of Run 1. The results of the model calibration are shown in Figure 4 (five days of Run 1). Figure 4 (b.1 and b.2) zoom in on the plot of the Wednesday data (48 to 72 h), and values of α_1 _are labelled. By using the proposed approach herein, the relative error (for the whole of Run 1) between experimental EC (29.8 g–C·m^−3^·h^−1^) and simulated EC (30.5 g–C·m^−3^·h^−1^) was improved by 2.3%. The model is able to better predict the dynamic variations in the outlet gas-phase emissions and in the dissolved carbon concentration in the recirculation tank than in previous calibration steps. 

### 3.2. Model Validation 

**Figure 5:**
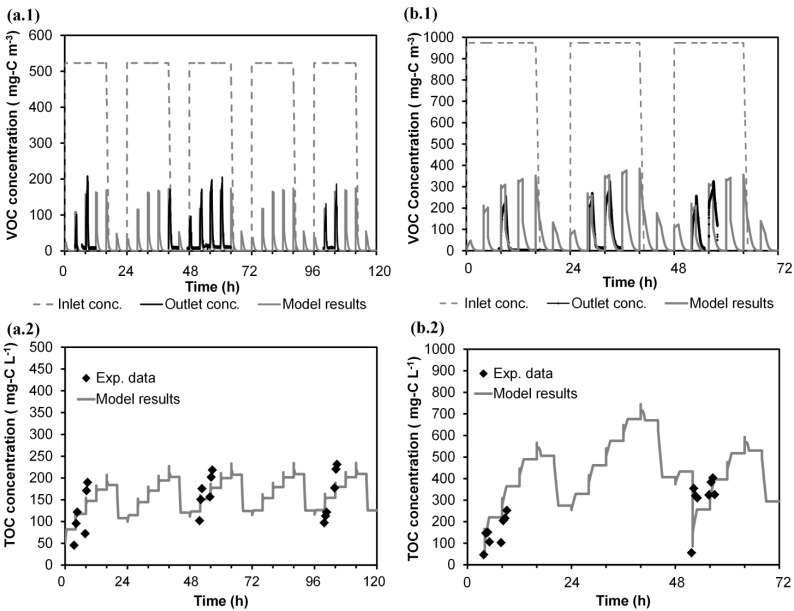
Weekly evolution of the experimental data and model predictions for the treatment of isopropanol by BTF (**a**) Run 2. (**a.1**) gas phase; (**a.2**) dissolved carbon in the water tank. (**b**) Run 3. (**b.1**) gas phase; (**b.2**) dissolved carbon in the water tank.

The mathematical model was validated by using two sets of independent experiments at the laboratory scale (Run 2 and Run 3, Table 1) to check the capability of the model to predict the evolution of the system over five days and three days, respectively. Experimental data along with the results of the model simulations are shown in Figure 5. The model is capable of reproducing the cyclical performance by using a different spraying duration (Run 2) or inlet load (Run 3). The relative error between experimental EC and simulated EC was 3.7% and 2.4% for Run 2 and Run 3, respectively. In both cases, the model shows a daily evolution of the outlet gas-phase concentrations: the gradual increase of the peaks when the air polluted with VOC is supplied to the BTF and its decrease when clean air is supplied; the model successfully predicts the available experimental data regarding outlet VOC emissions. The model also simulates the variations of the dissolved carbon in the water tank: the accumulation of dissolved carbon when the BTF is fed with VOC-polluted air and its decrease when clean air is used, showing good agreement with the available experimental TOC concentrations. For example, the model predicts the increase of the experimental organic carbon concentration from 55 (after purging the tank at 52 h) to ~400 mg–C·L^−1^ after the third daily spraying during Run 3. In spite of increasing the driving force, the intermediate purge had a negligible impact on the outlet VOC emissions (a similar experimental peak was obtained at the third daily spraying of Days 2 and 3). This corroborates that the gas-phase emission during spraying could be associated with the high resistance of the gas-liquid mass transfer. 

### 3.3. Model Simulations

**Figure 6:**
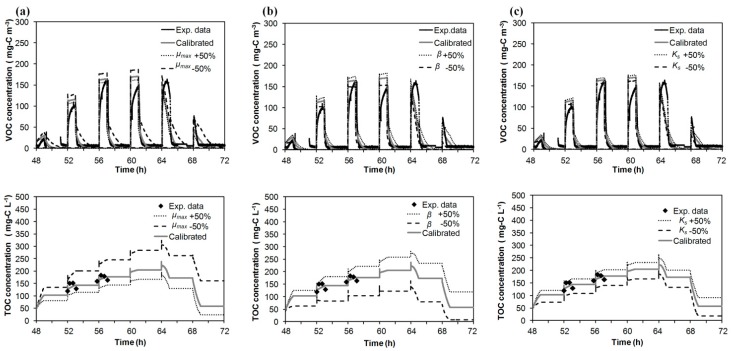
Effect of (**a**) *µ_max_*, (**b**) *β* and (**c**) *K_s_* on the outlet gas-phase concentration and on the dissolved carbon concentration in the recirculation tank. Experimental data correspond to the Wednesday data (48 to 72 h) of Run 1.

To assess the impact of the calibration parameters, *µ_max_*, *β* and *K_s_*, on the VOC outlet concentration and on the concentration of the dissolved carbon in the water tank, model simulations were carried out modifying by ±50% each parameter individually from the value listed in Table 3. The rest of the model parameters are listed in Table 3. Figure 6 shows the application of this study to Run 1 (Wednesday data, 48 to 72 h). When *µ_max_* was increased to 50%, the model predicted lower values in the maximum concentration of the gas phase during the spraying periods (161 mg–C·m^−3^). The predicted concentrations of the dissolved carbon in the water tank were lower (178 mg–C·L^−1^) than those obtained with the optimal value (224 mg–C·L^−1^) of *µ_max_*, while during the periods fed with clean air, the dissolved carbon concentration decreased faster (24 mg–C·L^−1^) than that obtained with the calibrated value (58 mg–C·L^−1^). In contrast, when the *µ_max_* was decreased by 50%, higher peaks were obtained during spraying periods (max peak 187 mg–C·m^−3^). It is important to note that the decreasing of the outlet concentration after a spraying was slower than the experimental decrease, indicating that the process was controlled by the kinetics. The dissolved carbon concentration was higher (320 mg–C·L^−1^) than that obtained with the optimal value of *µ_max_*. When *β* was increased up to 50%, greater concentrations in the gas and in the liquid phase were obtained (max values 182 mg–C·m^−3^ and 281 mg–C·L^−1^, respectively); while *β* was decreased by 50%, lower peaks in the outlet gas phase and a lower concentration in the water tank were achieved (max values 153 mg–C·m^−3^ and 140 mg–C·L^−1^, respectively). The parameter *β* is related to the transfer of pollutant and oxygen between liquid and biofilm, and this analysis shows that this parameter is one of the most sensitive in the modelling of the treatment of isopropanol by BTF. When *K_s_* was modified ±50%, the model appeared less sensitive to modifications of this parameter, obtaining a neglecting effect in the gas phase and less impact in the concentration of the dissolved carbon in the water tank than with the other parameters.

### 3.4. Model Application to Industrial Unit Processes

**Figure 7:**
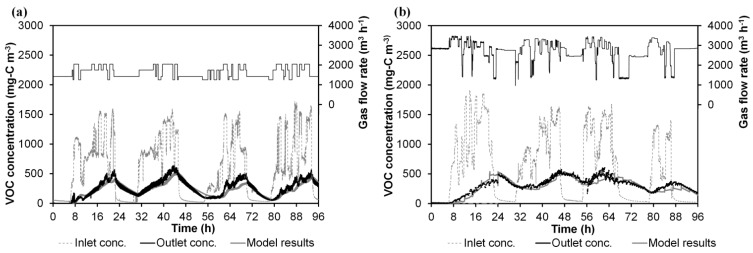
Experimental data and model results of the industrial BTF installed in a flexographic industry site: (**a**) Run 4 (**b**) Run 5.

The model proposed herein was applied by using experimental data from two different periods from an industrial BTF located at a flexographic industry site (Run 4 and Run 5). Experimental data from both runs have a two-year spacing. Table 4 shows the VOC composition of the industrial emission and the diffusion coefficient in water and Henry’s law constant for each compound that composed the VOC emission. Figure 7 shows the experimental data and the model simulations for both runs. In this figure, time 0 h refers to Monday 00:00 am. In contrast to those obtained in the laboratory experiments, the gaseous outlet emissions showed a buffered pattern, and no peaks associated with spraying or with instantaneous variations of the flow rate or inlet emissions were observed. During VOC inlet emissions coming from the factory (from 6:00 am to 12:00 pm), the outlet gas concentrations of the BTF were not clearly related to the spraying pattern; a continuous gradual increase of them was observed every day. During night periods (from 00:00 to 6:00 am), a slow decrease in the outlet emissions was observed; the VOC accumulated in the biofilm during periods receiving inlet emissions was not totally stripped or degraded over 6 hours, while when the bioreactor was operated with clear air for more than 24 h (weekends), the outlet gas concentration became negligible (data between 0 and 6 h in Figure 7). The industrial BTF was acting as an absorption-desorption system during VOC feeding-non-VOC feeding cycles. This seems to indicate that there is a difference in the rate-limiting steps between the treatment of hydrophilic VOC by biotrickling filtration at the industrial scale and for laboratory units. This finding was one of the challenges of this work. Based on these two observations (no peaks during spraying and continuous outlet emissions during non-VOC feeding periods), it was assumed that: (1) there was an extra mass transfer resistance between the gas and liquid phases during non-spraying periods not observed in the laboratory experiments; and (2) a thick biofilm was developed. 

**Table 4:** VOC composition and physical properties of the compounds of the industrial emission.

Compounds	Composition (%)	*D_P _(m^2^*·*s*^−^*^1^)* [25]	*H_P_* [27]
**Ethanol **	63	1.48 × 10^−9^	2.30 × 10^−4^
**Ethyl acetate**	22	9.57 × 10^−1^°	6.40 × 10^−3^
**1-Ethoxy-2-propanol **	13	8.49 × 10^−1^°	1.00 × 10^−6 *^

^*^ Data estimated from [30]

The simulation parameters for the application of the model to the industrial unit processes are listed in Table 3, except the overall mass transfer coefficients and the biofilm thickness. As the industrial BTF worked under conditions of variable gas velocities, the overall mass transfer coefficient for the pollutant was estimated for each simulated time point, applying the correlation proposed by San-Valero *et al.* [24], using the gas velocity and the weighted average value of Henry’s law constant of the VOC mixture (Equation (12)). This weighted average Henry’s law constant was calculated using the percentage composition of each compound and its Henry’s law constant (Table 4).

(12) $$K_{L}a_{P}=\frac{H_{P}}{3600}\left(11.59{\left(v_{G}3600\right)}^{0.85}\right)$$

The overall mass transfer of oxygen was experimentally determined in the laboratory, obtaining a value of 0.0066 s^−1^ for this packing material. The biofilm thickness (*δ*) was fitted to 500 µm in order to reproduce the slow desorption occurring during periods without supplying VOCs (every day from 0 am to 6 am). During spraying, it was assumed that there is similar mass transfer resistance to that in the laboratory systems (*α_1_* = 0.23). The emergence of the extra mass transfer resistance during non-spraying was related to the creation of a stagnant liquid phase. The switch parameter *α_2_* was set to one to consider the resistance to mass transfer within the gas-liquid interface during non-spraying periods. Simulation of both runs started with clean water (VOC accumulated in the water tank during working days was removed at the weekend). In the case of Run 5, the recirculation tank on Wednesday at 4 pm (64 h of Run 5 in Figure 7) was renewed with fresh water; this was included in the simulation. 

As can be seen in Figure 7, the correspondence between the calculated and the experimental VOC emissions is quite good. Relative errors between simulated and experimental data were 1.6% and 2.2% for Run 4 and Run 5, respectively. During the course of the biological process, VOCs were degraded, but a part of the load is cyclically absorbed-desorbed following the daily cycles of VOC feeding-non VOC feeding. The thick biofilm works as the sink (every day from 6 am to 12 pm) and source (every day from 0 am to 6 am) of the pollutant. The dynamic mathematical model approaches these phenomena through the existence of two resistances in series (gas-liquid, liquid-biofilm) that keep constant by operating the BTF with or without irrigation. This work appears as one of the first attempts to go more deeply into the modelling of the dynamic response associated with intermittent conditions (irrigation and inlet emissions), focusing on the observed differences between laboratory and industrial BTFs. In future work, the results of the model could be integrated into a software tool for the design and control of industrial BTFs. 

## 4. Conclusions 

A dynamic general model to simulate the removal of isopropanol emissions by biotrickling filtration has been developed. The model was built as a coupled set of equations for spraying and non-spraying periods in order to represent the intermittent irrigation usually performed in industrial applications. The model has been evaluated by using laboratory experiments working under intermittent spraying and intermittent VOC feeding to the bioreactor. The model predicts not only the overall performance, but also the outlet emission peaks occurring during spraying and the sharp decrease of gas-phase outlet concentration after spraying stops, indicating that mass transfer resistance during non-spraying was negligible. Practical applications of this model to predict the outlet VOC emissions in industrial BTF treating a mixture of pollutants were demonstrated. In this case, the thick biofilm and the mass transfer resistance between gas and stagnant liquid during non-spraying were the two main different characteristics from the model application to laboratory data. 

## Nomenclature

*a*: Specific surface area of the packing material (m^−1^)
*C*: Concentration (g·m^−3^)
*D*: Diffusion coefficient of substrates (m^2^·s^−1^)
*f(X_v_)*: Correction factor of diffusivity in biofilm according to Fan’s equation
*H*: Henry’s law constant
*K_s_*: Half saturation rate constants of the substrate (g–C·m^−3^)
*K_L_a*: Overall mass transfer coefficients of the substrates (s^−1^)
*M*: Number of divisions along the biofilm
*N*: Number of divisions along the column
*Q*: Flow rate (m^3^·s^−1^)
*S*: Concentration in the biofilm (g·m^−3^)
*t*: Time (s)
*v*: Superficial velocity calculated as a fraction of Q and S (m·s^−1^)
*V*: Volume (m^3^)
*x*: Coordinate for the depth in the biofilm, perpendicular to the biofilm surface
*X_v_*: Biomass concentration in the biofilm (g·m^−3^)
*Y*: Yield coefficient (g of dry biomass synthesized per g consumed)
*z*: Axial coordinate in the reactor
*Z*: Height of the reactor (m)

## Greek letters

*α_1_*: Correction of the mass transfer coefficient between biotic and abiotic systems
*α_2_*: Switch parameter of the model
*β*: Thickness of the liquid-biofilm interface (m)
*δ*: Thickness of the biofilm (m)
*θ_B_*: Volume fraction of the biofilm (–)
*θ_G_*: Porosity of the bioreactor (–)
*θ_L_*: Volume fraction of the liquid phase (–)
*θ_PM_*: Porosity of the packing material (–)
*µ_max_*: Maximum specific growth rate of the substratum (s^−1^)

## Subscripts

*i*: Substance (pollutant and oxygen)
*G*: Gas
*L*: Liquid
*B*: Biofilm
*P*: Pollutant
*O*: Oxygen
*R*: Reactor
*T*: Tank
